# High-Resolution MIMO Millimeter-Wave Radar Imaging Method for Non-Cooperative Targets

**DOI:** 10.3390/s26134106

**Published:** 2026-06-28

**Authors:** Jixing Guan, Junyu An, Guisheng Liao

**Affiliations:** 1 National Key Laboratory of Radar Signal Processing, Xidian University, Xi’an 710071, China; liaogs@xidian.edu.cn; 2 Information Science Academy, China Electronics Technology Group Corporation, Beijing 100086, China; anjunyu2026@163.com

**Keywords:** MIMO millimeter-wave imaging radar, array errors, super-resolution, BP imaging, concealed object detection

## Abstract

In the field of security screening imaging, millimeter-wave technology offers high imaging resolution and low radiation energy. However, it faces challenges such as difficulty in imaging non-cooperative moving targets, as well as bulky equipment and high costs. This paper proposes a high-resolution imaging method based on MIMO millimeter-wave radar. Firstly, the array model and slant range model are established, and a two-dimensional resolution scheme in range and height is constructed using a one-dimensional MIMO linear array and wideband signals. Then, the algorithm flow for MIMO millimeter-wave radar imaging is designed, and a range-domain super-resolution algorithm is introduced. This paper compensates for the phase coupling introduced by the transmitting array and target motion and successfully achieves two-dimensional imaging of non-cooperative targets based on the back-projection principle. Subsequently, the influence of array errors on the imaging results is analyzed. This method compensates for the phase coupling introduced by the transmit array and target motion and provides a theoretical analysis of array arrangement errors. Finally, the experimental results of the MIMO radar are analyzed. The final measured processing results show that the system can clearly reveal metal objects through cloth occlusion, and super-resolution processing yields sharper contours in the imaging of metal plates. Simulation analysis of imaging with array errors indicates that among the azimuth–elevation–range array position errors, the range array position error has a relatively significant impact.

## 1. Introduction

In the face of the current complex public security environment worldwide, particularly in high-density pedestrian areas such as aviation hubs and rail transit stations, traditional security screening methods are confronted with significant challenges. Therefore, imaging technology capable of efficiently screening concealed items carried on the human body, with non-invasive, non-stop, and covert characteristics, has become an urgent demand in the security field [1,2,3,4]. Although X-ray technology possesses imaging capability, X-ray-based detection methods are unsuitable for detecting concealed items on the human body due to excessive ionizing radiation, and they also raise privacy concerns. X-ray methods are primarily used for luggage screening [5,6,7]. Consequently, millimeter-wave and terahertz imaging technologies, owing to their high resolution, non-ionizing radiation, and ability to penetrate clothing, have been applied in imaging detection [8,9,10].

**Figure 1:**
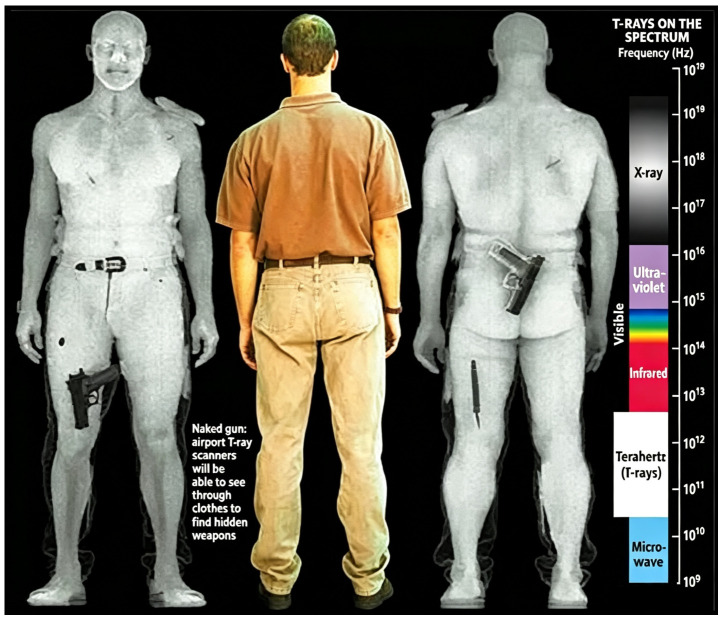
Three-dimensional security screening system image.

In millimeter-wave near-field imaging, achieving a sufficient signal-to-noise ratio within a limited observation time imposes extremely high requirements on the spatial scanning speed. Traditional mechanical scanning systems, constrained by physical inertia, suffer from low data refresh rates, are prone to imaging blur for dynamic targets, and thus fail to meet real-time imaging requirements [8,11,12]. In contrast, electronic scanning technology, with its microsecond-level beam switching speed, has become the ideal choice for achieving rapid echo accumulation. The two-dimensional multiple-input multiple-output (MIMO) millimeter-wave radar developed by Rohde and Schwarz (e.g., the QPS series) eliminates mechanical moving components. It innovatively employs a planar sparse array combined with time-division multiplexing (TDM) orthogonal waveform technology, successfully addressing the issues of imaging speed and resolution and achieving high-precision three-dimensional real-time imaging. The three-dimensional security screening imaging results are shown in Figure 1 [13]. However, due to the large number of channels required by the two-dimensional array configuration, the equipment cost is high. Furthermore, completing full-aperture TDM transmission requires a long cycle, which extends the effective accumulation time of the system. Additionally, in practical hardware systems, position errors of the array antenna elements are unavoidable. If these non-ideal factors are not properly analyzed and calibrated, they will lead to defocusing of imaging results and elevated sidelobe levels, thereby severely constraining the high-resolution imaging performance of the system [14,15,16]. Moreover, limited by the physical aperture size, the resolution of conventional algorithms is inherently constrained by the Rayleigh limit. It is difficult to break through the physical resolution bottleneck merely by increasing algorithm complexity, and this is often accompanied by high sidelobe levels, which are unfavorable for fine imaging of densely concealed objects.

To address the limitations of existing imaging technologies, this paper proposes a high-resolution imaging method for a non-cooperative target MIMO millimeter-wave radar. In the system architecture, an accurate array-target near-field slant range model is established. By leveraging wideband millimeter-wave signals and the virtual aperture synthesis technique of a MIMO linear array, the number of physical array elements is significantly reduced while achieving high-precision projection imaging in the range-elevation two-dimensional plane. Meanwhile, considering non-ideal factors in practical systems, an array error model is established, and the impact of array errors on imaging quality is analyzed. In the signal processing part, Doppler-domain compensation and Back-Projection (BP) algorithms are employed to achieve target focusing [17,18,19,20]. To further overcome the resolution limitation imposed by the physical aperture of the array, a super-resolution processing algorithm is introduced [21,22]. In the experimental part, tests are conducted on a metal plate wrapped in a fabric bag and on a moving scenario where a human body carries the concealed object. These tests confirm the radar system’s ability to detect metal objects through fabric obstruction and the improvement in imaging performance by super-resolution processing. Finally, simulation experiments are performed to analyze the impact of array errors on imaging.

## 2. MIMO Radar Array and Imaging Geometry Model

### 2.1. Array Model

**Figure 2:**
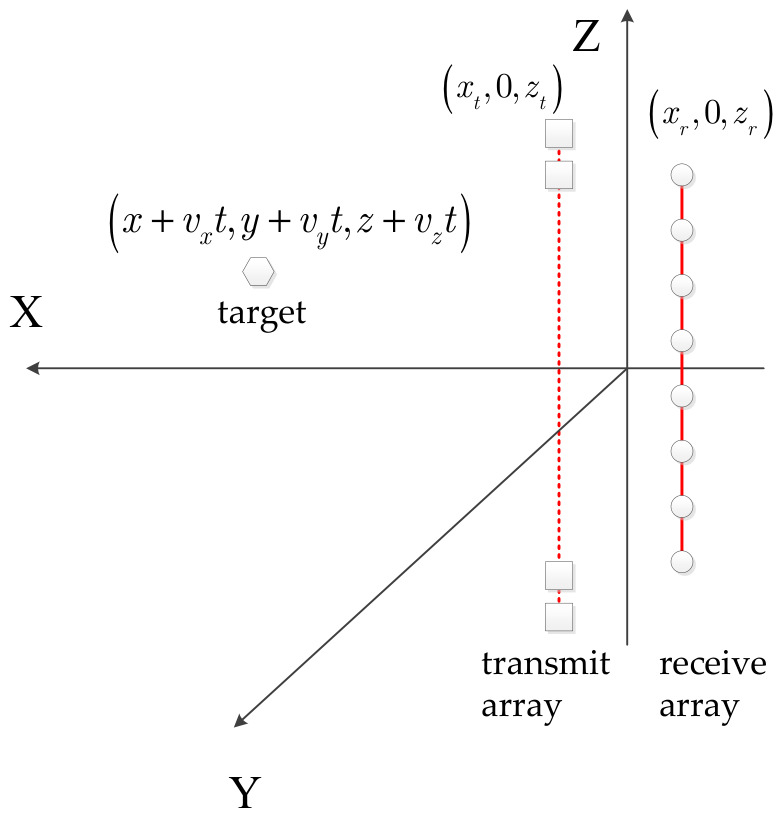
XOZ coordinate system array distribution model.

The structure of the transmit-receive linear array in this paper is shown in Figure 2. To describe the spatial positions of the array elements, a Cartesian coordinate system is established as follows: the antenna linear array is located in the XOZ plane, where the X-axis is defined as the azimuth direction, the Z-axis is defined as the elevation direction, and the Y-axis perpendicular to the array plane is defined as the range direction. The receiving elements are centrally arranged in the middle of the array, while the transmitting elements are divided into two groups symmetrically placed on both sides of the receiving array along the Z-axis. The transmit and receive arrays are not aligned in the X-direction [23]. Based on the above coordinate system, the mathematical models of the transmit and receive arrays can be expressed as $\left(x_{t},y_{t},z_{t}\right)$ and $\left(x_{r},y_{r},z_{r}\right)$, with the spacing between the transmit and receive arrays denoted as $d_{tr}$. The number of transmit array elements is set as $N_{tx}$ and $N_{tz}$, with spacings $d_{tx}$ and $d_{tz}$ in the X and Z directions, respectively. The number of receive array elements is $N_{rx}$ and $N_{rz}$, with spacings $d_{rx}$ and $d_{rz}$ in the X and Z directions, respectively.

In this paper, the spacing between the transmitter and receiver satisfies $d_{rz}=\frac{N_{tz}d_{tz}}{2}$. The array arrangement satisfies the following relationships:

(1) $$\{\begin{array}{c} x_{t}\;=\;-\frac{d_{tr}}{2}\\ z_{t}\;=\;\left[-\frac{\left(N_{rz}-1\right)N_{tz}}{4}+\left(-\frac{N_{tz}}{2}:-1\right),\frac{\left(N_{rz}-1\right)N_{tz}}{4}+\left(0:\frac{N_{tz}}{2}-1\right)\right]d_{tz}\\ x_{r}\;=\;\frac{d_{tr}}{2}\\ z_{r}\;=\;\frac{\left[-\frac{\left(N_{rz}-1\right)}{2}:\frac{\left(N_{rz}-1\right)}{2}\right]N_{tz}d_{tz}}{2} \end{array}$$

The Z-direction size of the receive sub-array $D_{z}$ and the Z-direction size of the transmit sub-array $D_{z}$ can be expressed as:

(2) $$D_{rz}=\frac{N_{rz}N_{tz}d_{tz}}{2},D_{tz}=\frac{N_{tz}d_{tz}}{2}$$

### 2.2. Slant Range Model

Based on the array model in Section 2.1, the radar received-signal slant-range model is established. The MIMO radar has a total of M transmitting elements that transmit sequentially in TDM mode, and N receiving elements receive signals simultaneously. The signal duration for each transmit element is $T_{r}$, and a coherent processing interval contains I complete transmission periods. Therefore, the total coherent integration time of the system can be expressed as $T_{i}\;=\;IMT_{r}$. The continuous time t can be expressed as the sum of three terms: the fast time $t_{r}$, the time for one transmission period $t_{m}$, and the slow time $t_{i}$:

(3) $$t\;=\;t_{r}\;+\;t_{m}\;+\;t_{i}\;=\;t_{r}\;+\;mT_{r}\;+\;iMT_{r}$$

where m (m = 0, 1, …, M−1) denotes the m-th transmit element, and i (i = 0, 1, 2, …, I−1) denotes the i-th transmission period.

The target coordinates are (x,y,z) and the velocity can be expressed as $\left(v_{x},v_{y},v_{z}\right)$. Then, the slant range can be expressed as:

(4) $$R\;=\;\frac{1}{2}\left[\begin{array}{c} \sqrt{{\left(x\;+\;v_{x}t\;-{\;x}_{t}\right)}^{2}\;+\;{\left(y\;+\;v_{y}t\;-\;y_{t}\right)}^{2}\;+\;{\left(z\;+\;v_{z}t\;-{\;z}_{t}\right)}^{2}}\\ +\sqrt{{\left(x\;+\;v_{x}t\;-\;x_{r}\right)}^{2}\;+\;{\left(y\;+\;v_{y}t\;-\;y_{r}\right)}^{2}\;+\;{\left(z\;+\;v_{z}t\;-\;z_{r}\right)}^{2}} \end{array}\right]\approx R_{tr}\;+\;vt$$

where $R_{tr}$ and v denote the initial slant range and the equivalent velocity of the target, respectively. Under this condition, the echo signal delay caused by target motion is expressed as:

(5) $$\tau \left(t_{r},t_{i},m,n\right)\;\approx \;\frac{2}{c}R\;=\;\frac{2}{c}\left[R_{tr}\;+\;v\left(t_{r}\;+\;t_{m}\;+{\;t}_{i}\right)\right]={\;\tau }_{im}+\frac{2}{c}vt_{r}$$

$\tau_{im}$ is the time delay considering the influences of array position and integration time. The slant range $R_{tr}$ between the target and the transmit-receive array can be further expressed as

(6) $$\begin{array}{ccc} R_{tr} & \approx & R_{0}-\frac{1}{2}\left[sin\theta_{x}\left(x_{t}+x_{r}\right)+sin\theta_{y}\left(y_{t}+y_{r}\right)+sin\theta_{z}\left(z_{t}+z_{r}\right)\right]\\ & + & \frac{1}{4R_{0}}\left[\begin{array}{c} {cos}^{2}\theta_{x}\left({x_{t}}^{2}+{x_{r}}^{2}\right)+{cos}^{2}\theta_{y}\left({y_{t}}^{2}+{y_{r}}^{2}\right)+{cos}^{2}\theta_{z}\left({z_{t}}^{2}+{z_{r}}^{2}\right)\\ -2\sin\theta_{x}\sin\theta_{z}\left(x_{t}z_{t}+x_{r}z_{r}\right)\\ -2\sin\theta_{y}\sin\theta_{z}\left(y_{t}z_{t}+y_{r}z_{r}\right)-2\sin\theta_{x}\sin\theta_{y}\left(x_{t}y_{t}+x_{r}y_{r}\right) \end{array}\right]\\ & + & \frac{1}{{4R_{0}}^{2}}\left\{\begin{array}{c} \sin\theta_{x}\cos^{2}\theta_{x}\left(x_{t}^{3}+x_{r}^{3}\right)+\sin\theta_{y}\cos^{2}\theta_{y}\left(y_{t}^{3}+y_{r}^{3}\right)\\ +\sin\theta_{z}\cos^{2}\theta_{z}\left(z_{t}^{3}+z_{r}^{3}\right)\\ +\left(1-3\;\sin^{2}\theta_{z}\right)\left[\sin\theta_{x}\left(x_{t}z_{t}^{2}+x_{r}z_{r}^{2}\right)+\sin\theta_{y}\left(y_{t}z_{t}^{2}+y_{r}z_{r}^{2}\right)\right]\\ +\left(1-3\;\sin^{2}\theta_{x}\right)\left[\sin\theta_{y}\left(x_{t}^{2}y_{t}+x_{r}^{2}y_{r}\right)+\sin\theta_{z}\left(x_{t}^{2}z_{t}^{2}+x_{r}^{2}z_{r}\right)\right]\\ +\left(1-3\;\sin^{2}\theta_{y}\right)\left[\sin\theta_{x}\left(x_{t}y_{t}^{2}+x_{r}y_{r}^{2}\right)\;+\sin\theta_{z}\left(y_{t}^{2}z_{t}^{2}+y_{r}^{2}z_{r}\right)\right]\\ -6\;\sin\theta_{x}\sin\theta_{y}\sin\theta_{z}\left(x_{t}y_{t}z_{t}+x_{r}y_{r}z_{r}\right) \end{array}\right\} \end{array}$$

Taking the geometric center of the antenna array as the observation point, $R_{0}=\sqrt{x^{2}+y^{2}+z^{2}}$ is defined as the slant range to the target, and $\theta_{x}$, $\theta_{y}$, and $\theta_{z}$ are angles describing the spatial direction of the target, which can be expressed as $\sin\theta_{x}=\frac{x}{R_{0}}$, $\sin\theta_{y}=\frac{y}{R_{0}}$, $\sin\theta_{z}=\frac{z}{R_{0}}$.

### 2.3. Imaging Geometry Model and MIMO Radar Parameter Design

**Figure 3:**
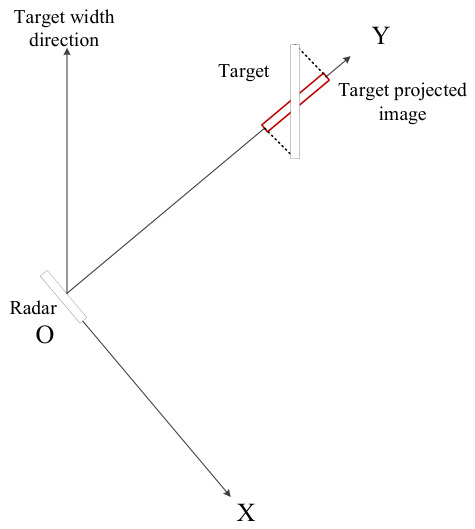
Imaging geometric relationship.

The system performs imaging in the YZ plane, using the signal bandwidth and array aperture to achieve range (Y) and elevation (Z) resolution, respectively. As shown in Figure 3, to obtain range-elevation imaging, the system employs squint imaging. The radar line of sight has a fixed squint angle with the target in the XY plane. By setting the squint observation angle, the target width feature is geometrically projected onto the range dimension. In principle, this is similar to the synthetic aperture radar (SAR) technique that projects the three-dimensional ground surface onto a two-dimensional plane.

The MIMO millimeter-wave radar has a height of 1.2 m and a center frequency of 95 GHz. The system employs a 10 GHz bandwidth signal, ensuring a range resolution of 0.02 m under a range broadening factor of 1.2. The equivalent aperture size in the elevation direction must be no less than 1.26 m, to ensure an elevation resolution of 0.015 m under an elevation broadening factor of 1.2. To optimize sidelobe performance, an 8Tx/16Rx MIMO architecture is adopted, with a Tx spacing of 1.1 cm and an Rx spacing of 4.4 cm. Through virtual aperture synthesis, 128 equivalent elements are obtained, with a total equivalent physical aperture of 1.4 m [4]. The imaging distance from the radar to the target is 5 m, and the imaging area size is 0.5 m.

## 3. Signal Processing Algorithm

Non-cooperative targets exhibit complex non-rigid motion and micro-Doppler effects, which affect radar imaging. First, the motion of non-cooperative targets causes image defocusing. Moreover, different parts of the target move at different velocities, making it impossible to perform imaging using a single set of parameters. To mitigate defocusing induced by target velocity, the data acquisition time, also known as integration time, is optimally designed as elaborated in Section 5. By using an extremely short integration time, the influence of target velocity within this interval can be neglected. To handle the spatial variation in velocity, a MIMO millimeter-wave imaging algorithm is proposed. By transforming the signal into the Doppler domain, different velocity components are effectively distinguished. BP imaging is then performed separately for each velocity to obtain images of target regions with distinct motion speeds. Finally, the overall image is reconstructed by superimposing the images corresponding to different velocities.

**Figure 4:**
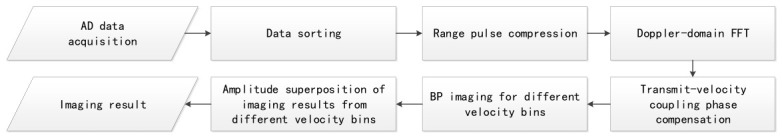
MIMO millimeter-wave radar imaging algorithm flow.

The MIMO millimeter-wave radar imaging algorithm flow is shown in Figure 4, with specific implementation detailed in Ref. [4]. First, the MIMO received signals are sorted, followed by range pulse compression to achieve range focusing. The range pulse compression can be replaced by the IAA algorithm to implement super-resolution processing. Then, the signals are transformed into the Doppler domain, and the transmit-velocity coupling phase compensation is performed for different target velocities. Finally, BP imaging is conducted for individual velocity cells, and the amplitude of the BP imaging results from different velocity cells is superimposed. The imaging results can be written as

(7) $$S=MNT_{r}T_{i}sinc\left[T_{r}\left(f_{r}-K_{r}\tau_{im}+f_{v}\right)\right]sinc\left[T_{i}\left(f_{i}-f_{v}\right)\right]$$

where $f_{r}$ denotes the range frequency, $K_{r}$ represents the linear frequency modulation rate of the signal, $f_{v}$ is the Doppler frequency induced by target velocity, and $f_{i}$ stands for the Doppler frequency.

In the MIMO millimeter-wave radar imaging algorithm flow, range-domain FFT is performed to achieve range focusing. However, the resolution of FFT is physically limited. If the spacing between two targets is smaller than the range resolution, a broad peak will form in the FFT spectrum, and the radar cannot distinguish the two targets. Therefore, when the signal bandwidth is limited, super-resolution methods are needed to improve the range resolution. This paper adopts the Iterative Adaptive Approach (IAA). IAA is an adaptive iterative algorithm based on weighted least squares, which performs spectral estimation using weighted least squares as the cost function. It constructs the signal covariance matrix from the spectral estimation results of the previous iteration and uses its inverse as the weighting matrix in the weighted least squares solution. IAA does not require prior knowledge of the number of signal sources. It can achieve super-resolution effectively with only a few or even a single snapshot. In the imaging flow described above, the range FFT is replaced by IAA super-resolution. The range-domain data for all receive channels and all accumulated pulses in the three-dimensional range-accumulation-receive channel data is processed with IAA. The range-domain data is defined as $x\in C^{N_{s}\times 1}$, where $N_{s}$ is the number of range points. The range scanning steering matrix is defined as $A\left(R\right)=\left[a\left(R_{1}\right),a\left(R_{2}\right),\cdots ,a\left(R_{l}\right),\cdots ,a\left(R_{L}\right)\right],l=1,\cdots ,L$, $a\left(R_{l}\right)={\left[1,e^{\frac{j2\pi K_{r}2R_{l}}{cf_{s}}},\cdots ,e^{\frac{j2\pi K_{r}\left(N_{s}-1\right)2R_{l}}{cf_{s}}}\right]}^{T}$, where L is the total number of scanning grid points and $f_{s}$ is the sampling rate. $K_{r}$ is the chirp rate.

The algorithm procedure is as follows:

Initialization: Calculate the initial complex amplitude for the *l*-th scanning range according to:

$\alpha_{l}^{\left(0\right)}=\frac{a^{H}\left(R_{l}\right)x}{a^{H}\left(R_{l}\right)a\left(R_{l}\right)}$ to obtain *L* initial power vector elements $p^{\left(0\right)}={\left[{\left|\alpha_{1}^{\left(0\right)}\right|}^{2},{\left|\alpha_{2}^{\left(0\right)}\right|}^{2},\cdots ,{\left|\alpha_{l}^{\left(0\right)}\right|}^{2},\cdots ,{\left|\alpha_{L}^{\left(0\right)}\right|}^{2}\right]}^{T}$.

Iteration: Compute the covariance matrix for the q-th (q ≤ Q, where Q is the maximum number of iterations) iteration $R^{q}=A\left(R\right)diag\left(p^{\left(q-1\right)}\right)A^{H}\left(R\right)$. Compute the complex amplitude for the l-th scanning range $\alpha_{l}^{\left(q\right)}=\frac{a^{H}(R_{l})(R^{q}{)}^{-1}x}{a^{H}(R_{l})(R^{q}{)}^{-1}a(R_{l})}$, to obtain the initial power vector $p^{\left(q\right)}\;=\;{\left[{\left|\alpha_{1}^{\left(q\right)}\right|}^{2},{\left|\alpha_{2}^{\left(q\right)}\right|}^{2},\cdots ,{\left|\alpha_{l}^{\left(q\right)}\right|}^{2},\cdots ,{\left|\alpha_{L}^{\left(q\right)}\right|}^{2}\right]}^{T}$. Iterate until the power vector converges or the maximum number of iterations is reached. The complex amplitude values for all scanning ranges from the last iteration are obtained. Subsequently, Doppler-domain FFT and other imaging processes are performed.

## 4. Integration Time Design

According to Equations (5) and (7), the displayed distance of the target is given by

(8) $$R_{s}=R_{tr}+v\left(t_{i}+\;t_{m}\right)+\frac{2vT_{r}}{\lambda }\rho_{r}$$

where $\rho_{r}$ is the range resolution. Therefore, the displayed distance of the target is related not only to the target position and array position but also to the target velocity, integration time, and transmission time. If the influence of the latter factors is too significant, it leads to a range error, which in turn causes errors in the phase compensation during subsequent BP imaging, resulting in image defocusing. Therefore, it is necessary to constrain the range walk caused by the latter factors to be less than half a range bin.

(9) $$v\left[\left(t_{i}+\;t_{m}\right)+2T_{r}\rho_{r}/\lambda \right]\;\leq {\;\rho }_{r}/2$$

Given $t_{i}\;+\;t_{m}\;<\;T_{i}\;=\;IMT_{r}$, the integration time satisfies

(10) $$T_{i}\;\leq \;\frac{\rho_{r}}{2v\left[1+2\rho_{r}/\left(\lambda MI\right)\right]}$$

If the range resolution is 1.5 cm and the target velocity is approximately 1 m/s, the integration time shall be less than 7.5 ms. The integration time of the system is designed as 3.2 ms; thus, the influence of human velocity can be neglected within the integration time.

## 5. Array Error Analysis

In this paper, the ideal array arrangement satisfies Equation (1), but in practice, deviations from Equation (1) exist due to errors. For Equation (6), under ideal conditions where $y_{t0}\;={\;y}_{r0}\;=\;0,x_{t0}\;+\;x_{r0}\;=\;0$, the expression can be simplified as:

(11) $$\begin{array}{ccc} R_{tr_{0}} & \approx & R_{0}-\frac{1}{2}\left[sin\theta_{z}\left(z_{t0}+z_{r_{0}}\right)\right]\\ & + & \frac{1}{4R_{0}}\left[{cos}^{2}\theta_{x}\left(x_{t0}^{2}+x_{r_{0}}^{2}\right)+{cos}^{2}\theta_{z}\left(z_{t0}^{2}+z_{r_{0}}^{2}\right)-2sin\theta_{x}sin\theta_{z}\left(x_{t0}z_{t0}+x_{r_{0}}z_{r_{0}}\right)\right]\\ & + & \frac{1}{4R_{0}^{2}}\left\{\begin{array}{c} \sin\theta_{x}{cos}^{2}\theta_{x}\left(x_{t0}^{3}+x_{r_{0}}^{3}\right)+\sin\theta_{z}{cos}^{2}\theta_{z}\left(z_{t0}^{3}+z_{r_{0}}^{3}\right)\\ +\left(1-3{sin}^{2}\theta_{x}\right)\sin\theta_{x}\left(x_{t0}^{2}z_{t0}+x_{r_{0}}^{2}z_{r_{0}}\right)+\left(1-3{sin}^{2}\theta_{z}\right)\sin\theta_{z}\left(x_{t0}^{2}z_{t0}+x_{r_{0}}^{2}z_{r_{0}}\right) \end{array}\right\} \end{array}$$

### 5.1. Ideal Array Slant Range History

If the array is ideal, the apparent range of the target is:

(12) $$R_{s0}\left(m,n\right)\;=\;R_{tr0}\;-\;\frac{f_{v}c}{2K_{r}}\;\approx {\;R}_{tr0}$$

The distance from the grid point to the array elements is calculated based on the grid position and the ideal transmit-receive positions, which can be expressed as:

(13) $$\begin{array}{c} R_{g}\left(m,n\right)=R_{g0}-\frac{1}{2}sin\theta_{\;gz}\left(z_{\;t0}+z_{\;r0}\right)\;+\;\frac{1}{4R_{g0}}\left[{cos}^{2}\theta_{\;gz}\left(z_{\;t0}^{\;2}\;+\;z_{\;r0}^{\;2}\right)+\left(x_{\;t0}^{\;2}\;+\;x_{\;r0}^{\;2}\right)\right]\\ +\frac{1}{4R_{g0}^{2}}\left[sin\theta_{\;gz}{cos}^{2}\theta_{\;gz}\left(z_{\;t0}^{\;3}+z_{\;r0}^{\;3}\right)\;+\;sin\theta_{\;gz}\left(x_{\;t0}^{\;2}z_{\;t0}+x_{\;r0}^{\;2}z_{\;r0}\right)\right] \end{array}$$

It is always possible to find a point on the grid satisfying $R_{g0}=R_{0}$ and $\theta_{gz}=\theta_{z}$. If the echo is also from an ideal array, then $R_{s}\left(m,n\right)\approx R_{g}\left(m,n\right)$, and the error between them can be expressed as:

(14) $$\begin{array}{ccc} ∆R_{0} & = & R_{g}\left(m,n\right)\;-\;R_{s_{0}}\left(m,n\right)\\ & = & \frac{1}{4R_{0}}\left[{sin}^{2}\theta_{x}\left(x_{t0}^{2}+x_{r0}^{2}\right)+2sin\theta_{x}sin\theta_{z}\left(x_{t0}z_{t0}+x_{r0}z_{r0}\right)\right]\\ & - & \frac{1}{4R_{0}^{2}}\left[\begin{array}{c} \sin\theta_{x}{cos}^{2}\theta_{z}\left(x_{t0}^{3}+x_{r0}^{3}\right)\\ +\left(1-3{sin}^{2}\theta_{z}\right)\sin\theta_{x}\left(x_{t0}z_{t0}^{2}+x_{r0}z_{r0}^{2}\right)\\ -3{sin}^{2}\theta_{x}\sin\theta_{z}\left(x_{t0}^{2}z_{t0}+x_{r0}^{2}z_{r0}\right) \end{array}\right] \end{array}$$

### 5.2. Error Array Slant Range History Error

If the array positions contain errors, the apparent range corresponding to the target is:

(15) $$R_{s}\left(m,n\right)\;=\;R_{tr}-\frac{f_{v}c}{2K_{r}}\;\approx \;R_{tr}$$

where $R_{tr}$ is given in Equation (10). If the array errors are expressed as:

(16) $$\begin{array}{c} \{\begin{array}{c} x_{t}={\;x}_{t0}\;+\;\Delta x_{t}\\ y_{t}=\;\Delta y_{t}\\ z_{t}={\;z}_{t0}\;+\;\Delta z_{t} \end{array}\;\;\;\;\{\begin{array}{c} x_{r}={\;x}_{r0}\;+\;\Delta x_{r}\\ y_{r}=\;\Delta y_{r}\\ z_{r}=\;z_{r0}\;+\;\Delta z_{r} \end{array}\\ x_{t}\;+\;x_{r}\;=\;\Delta x_{t}+\Delta x_{r} \end{array}$$

then the error between $R_{s}\left(m,n\right)$ and $R_{s}\left(m,n\right)$ can be expressed as:

(17) $$\begin{array}{ccc} ∆R & = & R_{g}\left(m,n\right)-R_{s}\left(m,n\right)\\ & \approx & ∆R_{0}+\frac{1}{2}\left[\begin{array}{c} \sin\theta_{z}\left(∆z_{t}+∆z_{r}\right)+\sin\theta_{x}\left(∆x_{t}+∆x_{r}\right)\\ +\sin\theta_{y}\left(∆y_{t}+∆y_{r}\right) \end{array}\right]\\ & + & \frac{1}{4R_{0}}\left[\begin{array}{c} -{cos}^{2}\theta_{x}\left(2x_{t0}∆x_{t}+2x_{r0}∆x_{r}\right)\\ +2\sin\theta_{y}\sin\theta_{z}\left(∆y_{t0}z_{t}+∆y_{r}z_{r0}\right)\\ -{cos}^{2}\theta_{z}\left(2z_{t0}∆z_{t}+2z_{r0}∆z_{r}\right)\\ +2\sin\theta_{x}\sin\theta_{y}\left(x_{t0}∆y_{t}+x_{r0}∆y_{r0}\right) \end{array}\right]\\ & + & \frac{1}{4R_{0}^{2}}\left\{\begin{array}{l} -sin\theta_{z}{cos}^{2}\theta_{z}\left(3z_{t0}^{2}∆z_{t}+3z_{r0}^{2}∆z_{r}\right)\\ -sin\theta_{z}\left(2x_{t0}z_{t0}∆x_{t}+2x_{r0}z_{r0}∆x_{r}+x_{t0}^{2}∆z_{t}+x_{r0}^{2}∆z_{r}\right)\\ -\left(1-3{sin}^{2}\theta_{x}\right)\left[sin\theta_{y}\left(x_{t0}^{2}∆y_{t}+x_{r0}^{2}∆y_{r}\right)\right]\\ +6sin\theta_{x}sin\theta_{y}sin\theta_{z}\left(x_{t0}∆y_{t}z_{t0}+x_{r0}∆y_{r}z_{r0}\right) \end{array}\right\} \end{array}$$

The additional error caused by the array position difference can be decomposed into three components, expressed as:

(18) $$\begin{array}{lll} ∆R & = & ∆R_{x}+∆R_{y}+∆R_{z}\\ ∆R_{r} & = & \frac{1}{2}\sin\theta_{x}\left(∆x_{t}+∆x_{r}\right)-\frac{1}{2R_{0}}{cos}^{2}\theta_{x}\left(x_{t0}∆x_{t}+x_{r0}∆x_{r}\right)\\ & - & \frac{1}{2R_{0}^{2}}\sin\theta_{z}\left(x_{t0}z_{t0}∆x_{t}+x_{r0}z_{r0}∆x_{r}\right)\\ ∆R_{y} & = & \frac{1}{2}\sin\theta_{y}\left(∆y_{t}+∆y_{r}\right)+\frac{1}{2R_{0}}\left[\begin{array}{c} \sin\theta_{y}\sin\theta_{z}\left(∆y_{t0}z_{t0}+∆y_{r}z_{r0}\right)\\ +\sin\theta_{x}\sin\theta_{y}\left(x_{t0}∆y_{t0}+x_{r0}∆y_{r0}\right) \end{array}\right]\\ & + & \frac{1}{4R_{0}^{2}}\{-\left(1-3{sin}^{2}\theta_{x}\right)\left[sin\theta_{y}\left(x_{t0}^{2}∆y_{t}+x_{r0}^{2}∆y_{r}\right)\right]\\ & + & 6\sin\theta_{x}\sin\theta_{y}\sin\theta_{z}\left(x_{t0}∆y_{t}z_{t0}+x_{r0}∆y_{r}z_{r0}\right)\}\\ ∆R_{z} & = & \frac{1}{2}\sin\theta_{z}\left(∆z_{t}+∆z_{r}\right)-\frac{1}{2R_{0}}{cos}^{2}\theta_{z}\left(z_{t0}∆z_{t}+z_{r0}∆z_{r}\right)\\ & + & \frac{1}{4R_{0}^{2}}\{\begin{array}{c} -\sin\theta_{z}{cos}^{2}\theta_{z}\left(3z_{t0}^{2}∆z_{t}+3z_{r0}^{2}∆z_{r}\right)\\ -\sin\theta_{z}\left(x_{t0}^{2}∆z_{t}+x_{r0}^{2}∆z_{r}\right) \end{array}\} \end{array}$$

In this paper, $R_{0}\;=\;5$, $x_{t0},x_{r0}\;\leq \;0.05$, $z_{t0},z_{r0}\;\leq \;0.36$, $\theta_{x},\theta_{y}\;\leq {\;30}^{{}^{\circ}}$, and $\theta_{z}\approx 90^{{}^{\circ}}$. Therefore, the above errors can be approximated as:

(19) $$\begin{array}{c} \Delta R\;=\;\Delta R_{x}\;+\;{∆R}_{y}\;+\;\Delta R_{z}\\ \Delta R_{x}\;=\;\frac{1}{2}\sin\theta_{x}\left(\Delta x_{t}\;+\;\Delta x_{r}\right)\\ \Delta R_{y}\;=\;\frac{1}{2}\sin\theta_{y}\left(\Delta y_{t}\;+\;\Delta y_{r}\right)\\ \Delta R_{z}\;=\;\frac{1}{2}\sin\theta_{z}\left(\Delta z_{t}\;+\;\Delta z_{r}\right) \end{array}$$

Among these, the Y-direction error has the largest impact. The resulting phase error is:

(20) $$\phi \;=\;\frac{4\pi }{\lambda }sin\theta_{y}\Delta y$$

If the error takes the form of a quadratic phase error, the requirement that the phase error $\leq \frac{\pi }{4}$ leads to the position error requirement:

(21) $$\Delta y\;\leq \;\frac{\lambda }{16sin\theta_{y}}\;\approx \;\frac{\lambda }{16}$$

In this paper, the wavelength is approximately 0.0032 m = 3.2 mm, and the position accuracy requirement is 0.02 mm. If the error takes the form of a random error with standard deviation $\sigma_{y}$, the resulting Integrated Side-Lobe Ratio (ISLR) increment is:

(22) $$\Delta ISLR\;={\;\left(\frac{4\pi }{\lambda }sin\theta_{y}\right)}^{2}\sigma_{y}^{2}\;\approx \;{\left(\frac{4\pi }{\lambda }\sigma_{y}\right)}^{2}$$

Therefore, if the ideal ISLR = −23.6 dB and the error-induced ISLR increment ≤ 0.5 dB, the requirement is $\sigma_{y}$ ≤ 0.01 mm.

## 6. MIMO Radar Imaging Experimental Results and Analysis

### 6.1. Metal Plate Imaging Analysis

**Figure 5:**
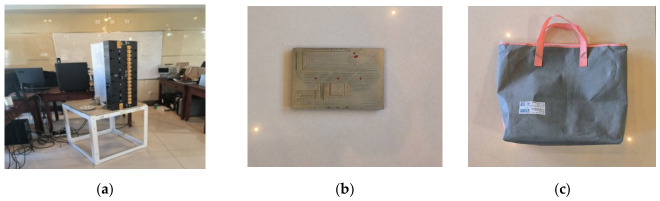
MIMO radar; imaging target; and obstruction. (**a**) MIMO radar prototype; (**b**) metal plate; and (**c**) non-woven fabric bag.

Based on the above analysis, Figure 5a shows the actual radar prototype. The radar prototype is used for penetration imaging experiments. The metal plate shown in Figure 5b is the imaging target, and the metal plate is placed inside the non-woven fabric bag shown in Figure 5c. The specific configuration of the antenna arrays and target is illustrated in Figure 3. The radar linear array is placed along the Z-direction at the origin, the center of the target is set on the Y-axis at a distance of approximately 6 m from the radar, and the angle between the target orientation and the Y-axis is about 45°.

**Figure 6:**
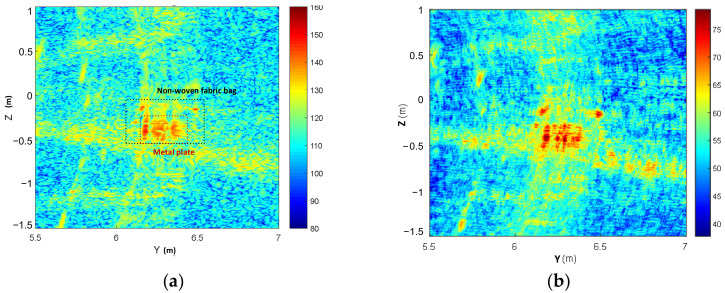
Metal plate imaging inside non-woven fabric. (**a**) Conventional processing image of the metal plate in the non-woven fabric bag; (**b**) range super-resolution processed image of the metal plate in the non-woven fabric bag.

The imaging results are shown in Figure 6. Figure 6a,b present the conventional processing image and the range super-resolution processed image, respectively. From Figure 6a, it can be observed that, except for the strong scattering characteristics exhibited by the bag opening zipper, the obstruction effect of the fabric bag is minimal. The outline of the internal metal plate is visible. The imaging algorithm accurately restored the spatial geometric position of the metal plate. From the range super-resolution processed imaging result in Figure 6b, compared with Figure 6a, the metal plate image has clearer edge contours. The left and right contours of the imaging result are sharper lines, and the edges are not blurred.

**Figure 7:**
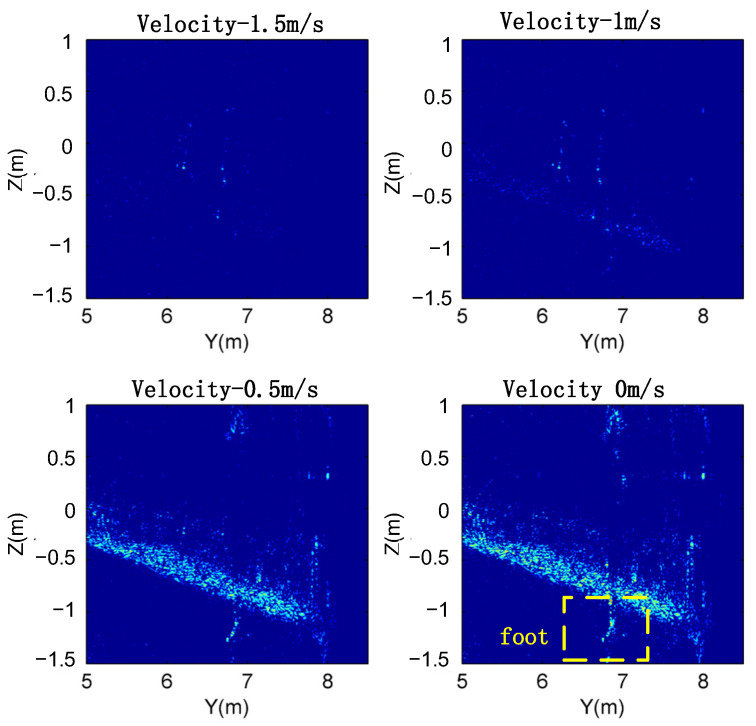

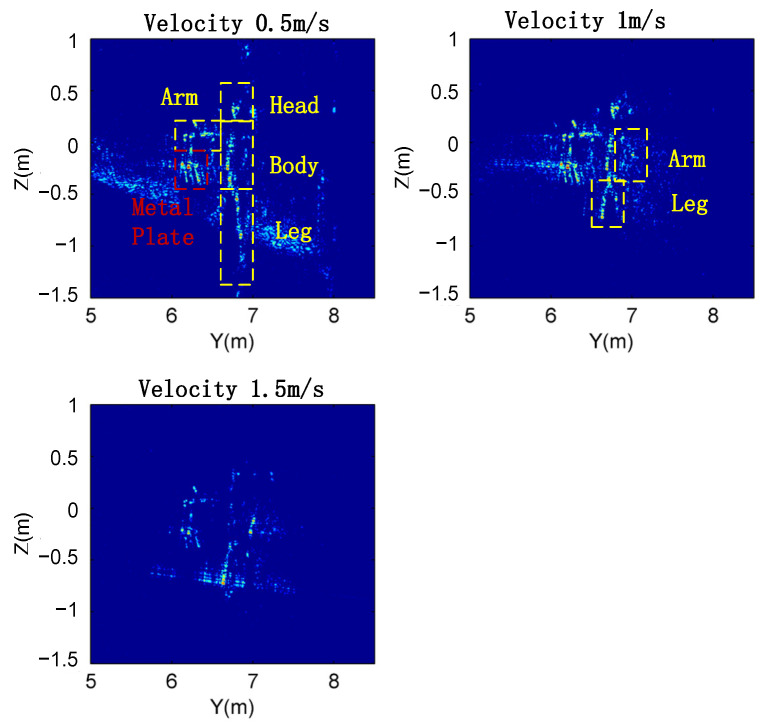
Imaging results of non-cooperative targets at different velocities.

In addition to static target imaging experiments, imaging experiments of non-cooperative targets are also carried out. In the experiment, a human held a metal plate contained in a non-woven fabric bag with his right arm outstretched horizontally and moved toward the radar. Given the varying velocities of different human body parts, the algorithm proposed in this paper firstly transforms the echo signals into the Doppler domain. Joint compensation of the Doppler frequency and transmit phase is then performed for targets at distinct velocities, followed by the back-projection (BP) algorithm to generate images corresponding to each velocity. The resulting imaging outputs are presented in Figure 7.

Figure 7 illustrates the imaging results of the non-cooperative target within the velocity range of −1.5 m/s to 1.5 m/s. It can be observed that different regions of the human body and the fabric bag exhibit distinct velocities, with dominant velocity components at 0 m/s, 0.5 m/s, and 1 m/s. At a velocity of 0 m/s, the outline of the left foot is distinguishable. Under the 0.5 m/s velocity condition, clear outlines are captured for the horizontally raised right arm, head, body, left leg, and the metal plate sealed inside the non-woven bag. When the velocity reaches 1 m/s, the outline of the head, left arm and both legs can be observed.

**Figure 8:**
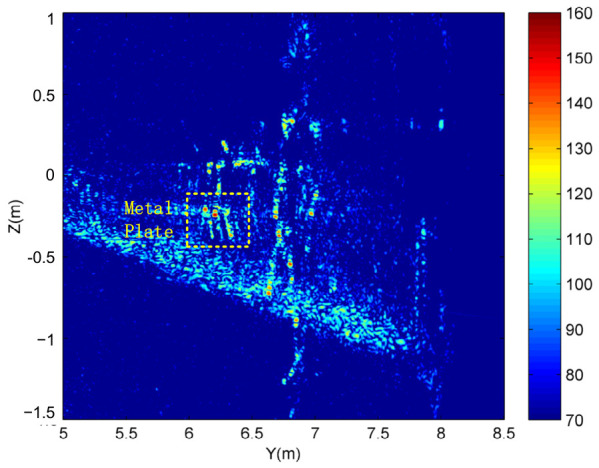
Imaging results of non-cooperative targets after multi-velocity fusion.

After generating images for all discrete velocities, image fusion across different velocities is implemented, and the fused outcome is displayed in Figure 8. Upon velocity-domain image fusion, all parts of the human body and the metal plate inside the non-woven bag are fully reconstructed. The metal plate is marked by the rectangular box in the figure. The plate is illustrated as a set of parallel lines, which differs from the uniform square shape obtained under the static imaging condition. This is due to the strong dependence of millimeter-wave radar imaging on the incident angle, whereby the imaging characteristics vary noticeably with changing incident angles.

### 6.2. Array Error Imaging Simulation Analysis

Based on the array error theoretical analysis, imaging simulations are performed. The target parameters are range 5 m, azimuth −0.3 m, and elevation −0.3 m.

#### 6.2.1. Elevation Error

**Figure 9:**
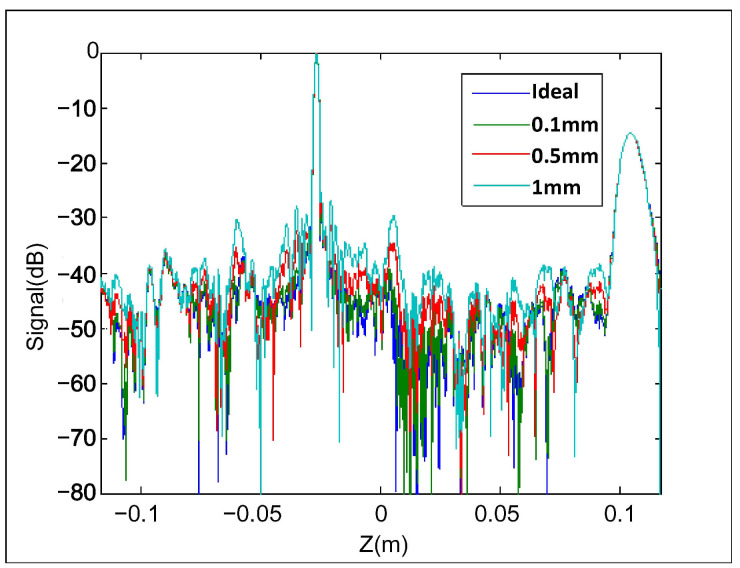
Elevation imaging results with elevation error.

The imaging results for different spacings are shown in Figure 9.

When errors exist in the elevation direction of the transmit elements, the imaging results and parameter summary are shown in Figure 9 and Table 1, respectively. The larger the elevation array position error, the greater the increments in the elevation ISLR and PSLR, and the imaging results show slight degradation compared to the ideal array imaging.

#### 6.2.2. Azimuth Error

**Figure 10:**
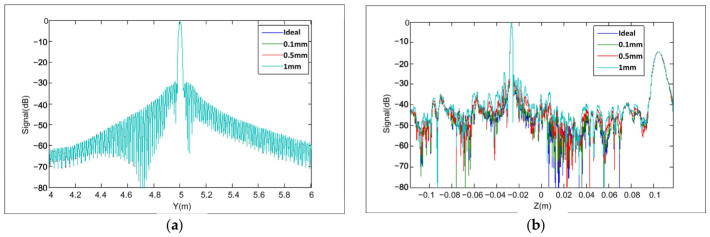
Imaging results with azimuth error. (**a**) Range imaging results; (**b**) elevation imaging results.

When errors exist in the azimuth direction of the transmit elements, the imaging results and parameter summary are shown in Figure 10 and Table 2, respectively. The larger the azimuth array position error, the greater the increments in the ISLR and PSLR of the elevation imaging results, and the imaging results show slight degradation compared to the ideal array imaging. The impact on range imaging results is negligible.

#### 6.2.3. Range Error

**Figure 11:**
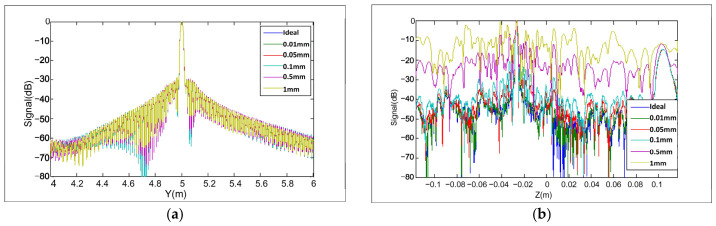
Imaging results. (**a**) Range imaging results; (**b**) elevation imaging results.

When errors exist in the range direction of the transmit elements, the imaging results and parameter summary are shown in Figure 11 and Table 3, respectively. The larger the range-direction array position error, the greater the increments in the ISLR and PSLR of the elevation imaging results. The elevation imaging results show significant degradation compared to the ideal array imaging. The impact on range imaging results is negligible.

#### 6.2.4. Summary

Based on the above analysis, among the azimuth, elevation, and range array positions, the range-direction array position error has the most significant impact. It primarily affects the elevation imaging quality, including signal amplitude, peak sidelobe, integrated sidelobe, and main lobe width. If the constraints require negligible resolution broadening and PSLR and ISLR degradation ≤ 0.5 dB, the position accuracy must be 0.01 mm.

## 7. Conclusions

This paper proposes a high-resolution MIMO millimeter-wave radar imaging method for non-cooperative targets and completes experimental validation. By utilizing the large bandwidth of millimeter waves and the elevation MIMO linear array, two-dimensional range-elevation imaging of targets is achieved, effectively addressing the existing problems of traditional mechanical scanning and electronic scanning technologies. To address the issue of low target imaging resolution, range super-resolution technology is introduced to break through the physical bandwidth limitation on range resolution. The theoretical impact of array errors on imaging results is analyzed. Experiments demonstrate that the radar system possesses excellent penetration detection capability. It can meet the requirements for rapid, non-contact security screening while reducing hardware costs, providing an effective solution for next-generation public safety screening technology. Through simulation analysis, this paper verifies the impact of array position errors on imaging, including signal amplitude, peak sidelobe, integrated sidelobe, and main lobe width, providing a theoretical basis for the calibration of practical systems.

## Figures and Tables

**Table 1:** Parameter summary of the elevation error effects.

Parameter	Value			
Tx elevation spacing (mm)	0	0.1	0.5	1
Signal amplitude (dB)	0	0	−0.030	−0.090
Range resolution (m)	1.6	1.6	1.6	1.6
Elevation resolution (m)	1.43	1.43	1.43	1.43
Elevation PSLR (dB)	−27.64	−27.43	−26.43	−25.02
Elevation ISLR (dB)	−23.63	−23.53	−21.11	−17.32
Elevation grating lobe (dB)	−36.94	−37.63	−34.39	−29.46

**Table 2:** Parameter summary of the azimuth error effects.

Parameter	Value			
Tx azimuth spacing (mm)	0	0.1	0.5	1
Signal amplitude (dB)	0	0	−0.02	−0.08
Range resolution (m)	1.6	1.6	1.6	1.6
Elevation resolution (m)	1.43	1.43	1.44	1.43
Elevation PSLR (dB)	−27.64	−27.43	−26.48	−24.27
Elevation ISLR (dB)	−23.63	−23.46	−21.07	−17.5
Elevation grating lobe (dB)	−36.94	−36.76	−35.58	−33.63

**Table 3:** Parameter summary of the range error effects.

Parameter	Value					
Tx range spacing (mm)	0	0.01	0.05	0.1	0.5	1
Signal amplitude (dB)	0	0	−0.05	−0.21	−4.86	−12.82
Range resolution (m)	1.6	1.6	1.6	1.6	1.6	1.6
Elevation resolution (m)	1.43	1.43	1.43	1.43	1.54	1.56
Elevation PSLR (dB)	−27.64	−27.33	−25.86	−20.73	−6.58	−0.35
Elevation ISLR (dB)	−23.63	−23.25	−18.91	−14.27	0.22	9.16
Elevation grating lobe (dB)	−36.94	−36.59	−34.13	−28.93	−12.28	−1.95

## Data Availability

The data supporting the findings of this study are not publicly available due to confidentiality restrictions.
